# Ribosomal Protein L13 Participates in Innate Immune Response Induced by Foot-and-Mouth Disease Virus

**DOI:** 10.3389/fimmu.2021.616402

**Published:** 2021-05-20

**Authors:** Junyong Guan, Shichong Han, Jin’en Wu, Yun Zhang, Manyuan Bai, Sahibzada Waheed Abdullah, Shiqi Sun, Huichen Guo

**Affiliations:** ^1^ State Key Laboratory of Veterinary Etiological Biology, Office International des Epizootie (OIE)/China National Foot-and-Mouth Disease Reference Laboratory, Lanzhou Veterinary Research Institute, Chinese Academy of Agricultural Sciences, Lanzhou, China; ^2^ School of Animal Science, Yangtze University, Jingzhou, China

**Keywords:** foot-and-mouth disease virus, ribosomal protein L13, virus-host interactions, antiviral immune response, virus antagonism

## Abstract

In addition to ribosomal protein synthesis and protein translation, ribosomal proteins also participate in tumorigenesis and tumor progression, immune responses, and viral replication. Here, we show that ribosomal protein L13 (RPL13) participates in the antiviral immune response induced by foot-and-mouth disease virus (FMDV), inhibiting FMDV replication. The overexpression of RPL13 promoted the induction and activation of the promoters of the nuclear factor-κB (NF-κB) and interferon-β (IFN-β) genes, and the expression and protein secretion of the antiviral factor IFN-β and proinflammatory cytokine interleukin-6 (IL-6). The knockdown of RPL13 had the opposite effects. We also found that the FMDV 3C^pro^ protease interacts with RPL13, and that its activity reduces the expression of RPL13, thus antagonizing the RPL13-mediated antiviral activity. This study extends our knowledge of the extraribosomal functions of ribosomal proteins and provides new scientific information on cellular antiviral defenses and virus-antagonizing mechanisms.

## Introduction

In eukaryotes, the ribosome is the site of protein biosynthesis in cells. The mature ribosome (80S) includes the large (60S) ribosomal subunit and the small (40S) ribosomal subunit. The 40S subunit contains 30 ribosomal proteins (RPs), whereas the 60S subunit contains 49 RPs. In normal cells, the ribosome biogenesis process is monitored in a complex and elaborate manner to ensure that the ribosomes are assembled accurately and function normally (1). However, the specific stimulation of cells including stimulation of chemical agents or radiation, lack of nutrients and deregulation of genes required for ribosome biogenesis often causes the dysfunction of RPs and regulatory proteins in the nucleolus, inducing ‘ribosomal stress’. Under these conditions, ribosome synthesis is blocked and a large number of free RPs accumulate in the nucleus. Under close cellular monitoring, the unstable RPs are rapidly degraded by ubiquitination. However, some ribosomal proteins evade those cellular regulatory processes and exert ribosome-independent functions, such as regulating the immune response (2).

Recent research has shown that some RPs are involved in the activation of the NF-κB signaling pathway. Ribosomal protein S3 (RPS3) is a subunit of NF-κB, and binds to RelA (p65), enhancing its DNA binding activity, thereby selectively promotes the transcription of certain cytokines downstream from NF-κB (3). At present, two kinds of signal pathways have been found to mediate the activation of NF-κB signal pathway regulated by RPS3. In one, stimulation by tumor necrosis factor-α (TNF-α) promotes the expression of cystathionine lyase (CSE), which mediates the sulfhydrylation of the p65 subunit of NF-κB, and in turn promotes the combination of p65 and RPS3 (4). In the other pathway, IKKβ mediates the phosphorylation of RPS3 at serine 209 (S209), causing RPS3 to enter the nucleus to promote the transcription of specific factors downstream from NF-κB (5). IKKβ also activates NF-κB by inducing the ubiquitination of IκB and its degradation by proteasomes. This indicates that the IKKβ–RPS3 cascade can be used as an alternative pathway to selectively activate the NF-κB signaling pathway. The knockdown of RPS27 blocks the phosphorylation of p65 at S536 and of IκBα at S32, inhibiting the entry of NF-κB into the nucleus and reducing its DNA-binding properties, thereby blocking the NF-κB signaling pathway (6). RPL13 interacts with retinoic inducible gene-I (RIG-I) and binds to the 3′ untranslated region (3′-UTR) of NF-κB1 mRNA to promote the translation of NF-κB1, thereby enhancing the activation of NF-κB and the expression of the downstream inflammatory genes (7).

The innate-immunity-mediated inflammatory response is an important defense response to infection, but an excessive inflammatory response causes tissue damage, so this process must be closely regulated. Different from RPL13, ribosomal protein L13A (RPL13A) associated with ribosomes but is not required for canonical ribosome function and may have more extra-ribosomal functions. Studies have shown that ribosomal protein L13A (RPL13A) can act as a negative regulator of the inflammatory response (8). In the late stage of interferon-γ (IFN-γ) stimulation, the IFN-γ-activated inhibitor of translation (GAIT) complex formed by RPL13A, glyceraldehyde-3-phosphate dehydrogenase (GAPDH), glutamyl-prolyl-tRNA synthetase (EPRS), and NS1-associated protein 1 (NSAP1), which selectively binds to the 3′-UTR of related inflammatory genes, inhibits the translation of related inflammatory genes. A delay in the formation of the GAIT complex ensures that the early inflammatory response eliminates the pathogen, and then blocks the excessive development of the inflammatory response to ensure that the inflammatory tissues return to normal (9).

The wide range of extraribosomal functions of the RPs has aroused widespread interest among virologists. In recent years, several RPs have been shown to regulate viral replication. Some RPs promote viral replication by mediating the internal ribosome entry site (IRES)-dependent translation mechanism. For example, RPS5, RPS6, RACK1, and RPS25 promote the binding of the 40S small subunit to IRES by binding to IRES in the genomic 5′ untranslated region (5′-UTR) of hepatitis C virus (HCV), thus, promoting the initiation of viral protein translation (10–13). The knockdown of RPS25 inhibits the replication of viruses that depend on the IRES for the translation of their proteins, including HCV, classical swine fever virus (CSFV), encephalomyocarditis virus (EMCV), poliovirus (PV), enterovirus 71 (EV71), and cricket paralysis virus (CrPV) (12). With the knockdown of RPS6, the abundance of free 40S ribosomal subunits decreases, which specifically inhibits HCV IRES-mediated translation (11). In contrast, RPL40 specifically promotes the cap-dependent translation of vesicular stomatitis virus (VSV) in the family *Rhabdoviridae*. RPs can also interact with specific viral proteins to regulate the process of viral replication. For example, the N protein of Hantavirus interacts specifically with RPS19, which promotes the binding of the 40S subunit to both the 5′ cap structure and a conserved triple sequence in the viral genomic 5′-UTR, and then participates in the initiation of viral protein translation (14, 15). The dengue virus (DENV) NS1 protein binds to RPL18, which then migrates to the perinuclear region 48 h after viral infection. When RPL18 is knocked down, the viral titer is significantly reduced (16). Epstein-Barr virus (EBV) nuclear antigen 1 (EBNA1) interacts with RPL4 to promote the transactivation of oriP-dependent transcription by EBNA1, thus establishing persistent infections of EBV (17). The overexpression of RPL9 promotes the entry into the cell nucleus of the Gag protein of mouse mammary tumor virus (MMTV), and the interaction between the Gag protein and RPL9 in the nucleus supports the assembly of MMTV particles (18).

Other RPs inhibit viral replication. As mentioned above, IFN-γ treatment induces RPL13A to participate in the formation of GAIT complex to inhibit the translation of related inflammatory genes in order to control excessive inflammation (9). Other studies have shown that respiratory syncytial virus (RSV) infection causes RPL13A to be released from the 60S large subunit. When RPL13A recognizes the specific hairpin structure in the mRNA 3′-UTR of RSV M protein, a virus-activated translation inhibition complex (VAIT) is formed, which inhibits the translation of the M protein and exerts antiviral functions (19). The interaction between the rabies virus (RABV) P protein and RPL9 promotes the translocation of RPL9 from the nucleus to the cytoplasm and inhibits the early transcription of RABV (20). RPL19 through participate in TLR3-receptor-mediated signaling pathways and promotes cytokine secretion to inhibit VSV replication (21).

Foot-and-mouth disease (FMD) is a highly contagious and economically devastating viral disease caused by a small non-enveloped RNA virus: Foot-and-mouth disease virus (FMDV) (22). FMDV infection is recognized by toll like receptor 2 (TLR2), TLR3, and TLR7 of the TLR family and melanoma differentiation-associated protein 5 (MDA5) of the retinoic acid inducible gene I (RIG-I) like receptors (RLRs) family, and this recognition initiates downstream signaling pathways, activates the corresponding transcription factors, and induces the expression of specific cytokines. Of these, type I IFN is the most important antiviral factor, and it has recently been reported that type III IFN also inhibits FMDV replication. FMDV infection mainly induces the activation of type I and type III IFNs by inducing the activation of interferon regulatory factors (IRFs) and NF-κB. Type I and type III IFNs activate the JAK/STAT pathway by combining pattern recognition receptors (PRRs), and induce the expression of various IFN-stimulated genes (ISGs), such as PKR, RNase L, ISG15, ISG54, ISG56, 2’,5’-OAS1, and MxA, and genes encoding other antiviral proteins. The encoded proteins act synergistically and play an important role in inhibiting FMDV replication (23). However, FMDV has evolved strategies to counteract or even destroy the host’s innate immune system in order to replicate, reproduce, and spread throughout its body (24). In this study, we show that in PK-15 cells, RPL13 mediates the antiviral immune response induced by FMDV to inhibit FMDV replication. To maintain its own replication, FMDV antagonizes RPL13-mediated resistance by degrading RPL13.

## Materials and Methods

### Cells and Virus

Baby hamster kidney cells [BHK-21, American Type Culture Collection (ATCC) CCL-10] and porcine kidney cells (PK-15, ATCC CCL-33) were maintained in Dulbecco’s modified Eagle’s medium (DMEM) (Gibco, CA, USA) containing 10% fetal bovine serum (FBS; Gibco), 100 U/ml penicillin, and 100 μg/ml streptomycin and cultured in an incubator at 37°C under 5% CO_2_. FMDV strain O/BY/CHA/2010 (GenBank accession no. JN998085.1) is stored by the OIE/National Foot-and-Mouth Disease Reference Laboratory (Lanzhou, China). FMDV was grown and passaged in BHK-21 cells, and the titer of FMDV was determined with a median tissue culture infective dose (TCID_50_) assay.

### Antibodies and Reagents

Anti-RPL13, anti-DDX3, and anti-G3BP1 monoclonal antibodies were purchased from Abcam (Cambridge, MA, USA). Anti-green fluorescent protein (GFP), anti-FLAG, and anti-β-actin monoclonal antibodies were purchased from Santa Cruz Biotechnology (CA, USA). Anti-hemagglutinin (HA) monoclonal antibody was purchased from Cell Signaling Technology (Danvers, MA, USA). Horseradish peroxidase (HRP)-labeled secondary antibodies were purchased from Sigma-Aldrich (St. Louis, MO, USA). Polyclonal pig antiserum directed against FMDV was prepared and stored in our laboratory. Lipofectamine LTX Reagent for the transfection of recombinant plasmids and Lipofectamine RNAiMax Reagent for the transfection of siRNA were purchased from Invitrogen (CA, USA). The QuikChange Site-Directed Mutagenesis Kit was purchased from Agilent Technologies (CA, USA). RNA was extracted with TRIzol Reagent, purchased from Invitrogen (CA, United States). The specific inhibitors of helicase DDX3, RK-33 and ketorolac salt, were purchased from Selleck Chemicals (Houston, TX, USA).

### RNA Immunoprecipitation and Reverse Transcription (RT)–PCR

PK-15 cells were infected with FMDV at a multiplicity of infection (MOI) of 1. At 5 h postinfection (hpi), the cells were treated with radioimmunoprecipitation assay (RIPA) buffer (Beyotime Biotechnology) containing a protease inhibitor and RNase inhibitor, and the supernatant of the cell lysate was collected. Protein A beads were mixed with the cell lysate, incubated on ice for 1 h, and then centrifuged at 1000 × g for 10 min at 4°C to remove the complexes nonspecifically bound to the protein A beads. Then 8 μl of anti-DDX3, anti-RPL13, or IgG antibody was added to the cell lysate, or no antibody was added as the negative control. The samples were rotated overnight at 4°C. The pretreated protein A beads were added and incubated for 2–4 h at 4°C. The mixture of immune complexes was collected by centrifugation at 1000 × g for 5 min at 4°C and washed three times with lysis buffer. The complexes were then resuspended and precipitated in 400 μl of buffer (100 mM Tris-HCl [pH 8.0], 12.5 mM EDTA, 150 mM NaCl, 1% SDS) containing albumin K and incubated for 30 min at 37°C. The total RNA was then extracted with TRIzol Reagent (Invitrogen, CA, USA) and an RT–PCR analysis, with the One Step RT–PCR Kit (Takara) was performed to detect the FMDV IRES, ORF, and 3′-UTR, and the RPS16 and GAPDH gene fragments. The sequences of the primers used in this experiment are shown in **Table 1**.

**Table 1:** Primers used in the RIPA assay.

Primers name	Sequence (5’-3’)	Amplified-Fragment Length	Target genes
IRES-Fwd	CACAGGTTCCCACAACCGACAC	455 bp	FMDV IRES
IRES-Rev	GCAGTGATAGTTAAGGAAAGGC		
3’UTR-Fwd	GTTGCTAGTGATTATGACTTGGAC	443 bp	FMDV 3D and 3’UTR
3’UTR-Rev	CTTACGGCGTCGCTCGCCTCAGAG		
pRPS16-Fwd	CTGCAGCCATGCCTTCCAAGGGT	463 bp	porcine RPS16
pRPS16-Rev	TCATCACGATGGGCTTATCGGT		
pGAPDH-Fwd	GTCCATGCCATCACTGCCACCCAG	333 bp	porcine GAPDH
pGAPDH-Rev	GCTGTTGAAGTCACAGGACACAAC		

### RNA Interference (RNAi)

When cells grown to 70% confluence, the cell culture medium was discarded and cell maintenance solution containing 2% FBS was added. The cells were transfected with small interfering RNAs (siRNAs) with Liposome RNAiMAX Reagent (Invitrogen) and incubated for 36–48 h. The siRNAs targeting the candidate genes and negative control (NC) siRNA were synthesized by GenePharma (Shanghai, China). The siRNA sequences for the target genes are shown in **Table 2**.

**Table 2:** The sequences of siRNAs targeting candidate genes.

Genes name	siRNA sequence
porcine RPL13	5’-GGAAUGGCAUGAUCCUGAA-3’
porcine DDX3	5’-GCAAAGAUUCACUAACCUU-3’
NC siRNA	5’-UUCUCCGAACGUGUCACGU-3’

### Plasmid Construction

The total RNA of PK-15 cells was extracted and used as the template from which to amplify the cDNA of RPL13 and DDX3. The cDNA fragments of pCMV-N-FLAG vector, RPL13 and DDX3 were digested by *Eco*RI and *Xho*I endonuclease, respectively, and the cDNA fragments of RPL13 and DDX3 were inserted into pCMV-N-FLAG skeleton vector to obtain FLAG-RPL13 and FLAG-DDX3 recombinant plasmids, respectively. The pEGFP-N1 vector (Clontech, USA) and RPL13 cDNA were both digested with *Xho*I and *Eco*RI endonuclease, and the digested RPL13 cDNA was inserted into the pEGFP-N1 skeleton vector to construct a plasmid expressing recombinant enhanced green fluorescent protein (EGFP)–RPL13. The cDNAs of FMDV nonstructural proteins L^pro^, 2B, 2C, 3A, 3B, 3C^pro^, and 3D^pol^ were cloned from the genome of FMDV strain O/BY/CHA/2010, and then inserted into the pCMV-N-FLAG vector. The 3C^pro^ cDNA was also inserted into the pCMV-N-HA vector. Several residues of HA–3C^pro^ were mutated with a site-directed mutagenesis kit (Agilent Technologies CA, USA) to generate HA–3C^pro^ mutants H46Y, H84N, C163G, and H205R. Bicistronic reporter plasmids pGL4-NF-κB-Luc, pGL4-IFN-β-luc, pRL-TK-Renilla-luc are provided by Prof. Shao-Bo Xiao at Huazhong Agricultural University, psiCHECK-FMDV were constructed as previously described (25). All the recombinant plasmids were verified with DNA sequencing.

### Immunoprecipitation Assay

PK-15 cells were cotransfected with the appropriate recombinant plasmids and after incubation for 24 h, the cells were collected and lysed with RIPA buffer containing a protease inhibitor. Each cell lysate was centrifuged at 15000 × g for 20 min at 4°C, and the supernatant was collected. An aliquot of the supernatant (50−100 μl) was reserved as the input sample. The appropriate antibody was added to the remaining supernatant, which was rotated at 4°C. The protein samples containing the incubated antibodies were added to pretreated protein G beads and incubated at 4°C for 2–4 h. The antibody–bead mixture was then centrifuged at 3000 × g for 30 s at 4°C and the supernatant was discarded. After the beads were washed 2–3 times with lysis buffer containing a protease inhibitor and reductant, 50 μl of 1 × SDS loading buffer was added and the samples were boiled in a metal bath for 5–10 min, before analysis with western blotting.

### Luciferase Reporter Assays

After PK-15 cells were knocked down or overexpressed, or PK-15 cells were treated with specific inhibitors, the cells were transfected with the corresponding bicistronic plasmid (pGL4-NF-κB-Luc, pGL4-IFN-β-luc, pRL-TK-Renilla-luc or psiCHECK-FMDV) respectively. After incubation for 24 h, the supernatants were discarded and the cells were washed with 1 × phosphate-buffered saline (PBS). The special cell lysate buffer for dual luciferase reporter assay was added, and the signal intensities of firefly luciferase (Fluc) and Renilla luciferase (Rluc) were detected with the Dual Luciferase Reporter Assay Kit (Promega) according to the manufacturer’s instructions.

### Quantitative RT–PCR (RT–qPCR)

PK-15 cells were transfected with FLAG–RPL13 or the empty FLAG vector and incubated for 24 h, or PK-15 cells were transfected with RPL13 siRNA or NC siRNA, and incubated for 48 h, and then infected with FMDV (MOI = 0.5). At 2, 4, 6, and 8 hpi, the total RNA in the cell lysates was extracted with TRIzol Reagent and reverse transcribed to cDNA. Quantitative PCR (qPCR) was then used to detect the expression levels of the target genes. The primer sequences are shown in **Table 3**.

**Table 3:** Primers used in the qPCR assay.

Primers name	Sequence (5’-3’)	Target genes
FMDV-Fwd	CAAACCTGTGATGGCTTCGA	FMDV 3D
FMDV-Rev	CCGGTACTCGTCAGGTCCA	
IFN-β-Fwd	GCTAACAAGTGCATCCTCCAAA	porcine IFN-β
IFN-β-Rev	AGCACATCATAGCTCATGGAAAGA	
IL-6-Fwd	CTGCTTCTGGTGATGGCTACTG	porcine IL-6
IL-6-Rev	GGCATCACCTTTGGCATCTT	
RPL13-Fwd	GAGTCATCACGGACGAGGAG	porcine RPL13
RPL13-Rev	TCCTGTTCTGCAGCTTCCTT	
hGAPDH-Fwd	GTCCATGCCATCACTGCCACCCAG	hamster GAPDH
hGAPDH-Rev	GCTGTTGAAGTCACAGGACACAAC	
pGAPDH-Fwd	ACATGGCCTCCAAGGAGTAAGA	porcine GAPDH
pGAPDH-Rev	GATCGAGTTGGGGCTGTGACT	
PKR-Fwd PKR–Rev	GGAAGAAAACAAACACAGCTTGAA CCAAATCCACCTGAGCCAATT	porcine PKR

### Western Blotting

After SDS-PAGE of the boiled protein samples, they were transferred to polyvinylidenfluoride (PVDF) membranes and blocked with 1 × Tris-buffered saline with Tween 20 (TBST) containing 5% skimmed milk for 1 h. The corresponding primary antibody (diluted 1:1000) was then incubated with the membrane overnight at 4°C. The membrane was washed four times with 1 × TBST on a shaker for 5 min each time. The membrane was then incubated with the corresponding HRP-labeled secondary antibody (diluted 1:4000). The membrane was washed four times with 1 × TBST on a shaker for 5 min each time. Finally, ECL chromogenic solution (Sigma) was added, then exposure.

### TCID_50_ Assay

Various proteins were knocked down or overexpressed in PK-15 cells, which were then infected with FMDV (MOI = 0.5). The infected cells (including the cell supernatant) were collected at different time points and freeze–thawed three times. The collected cell fluid was then serially diluted and added to a 96-well plate with eight wells per sample, at 100 μl per well. Then 100 μl of BHK-21 cell suspension (1.5 × 10^6^/ml) was added to each well and the cells were incubated at 37°C in a 5% CO_2_ incubator for about 70 h to determine the number of cytopathic holes and the TCID_50_ was calculated with the Reed–Muench method. Each datum is the mean result of three independent experiments.

### Enzyme-Linked Immunosorbent Assay (ELISA)

After the knockdown or overexpression of various proteins in PK-15 cells, the cells were infected with FMDV (MOI = 0.5), and the supernatants of the infected cells were collected at different time points. The levels of IFN-β and IL-6 in the cell supernatants were then determined according to the instructions of the porcine IFN-β QuantiKine ELISA kit (R&D system, CA, USA) and IL-6 QuantiKine ELISA kit (Novatein Biosciences, MA, USA).

## Results

### RPL13 and DDX3 Are Required When FMDV Infects PK-15 Cells

**Figure 1:**
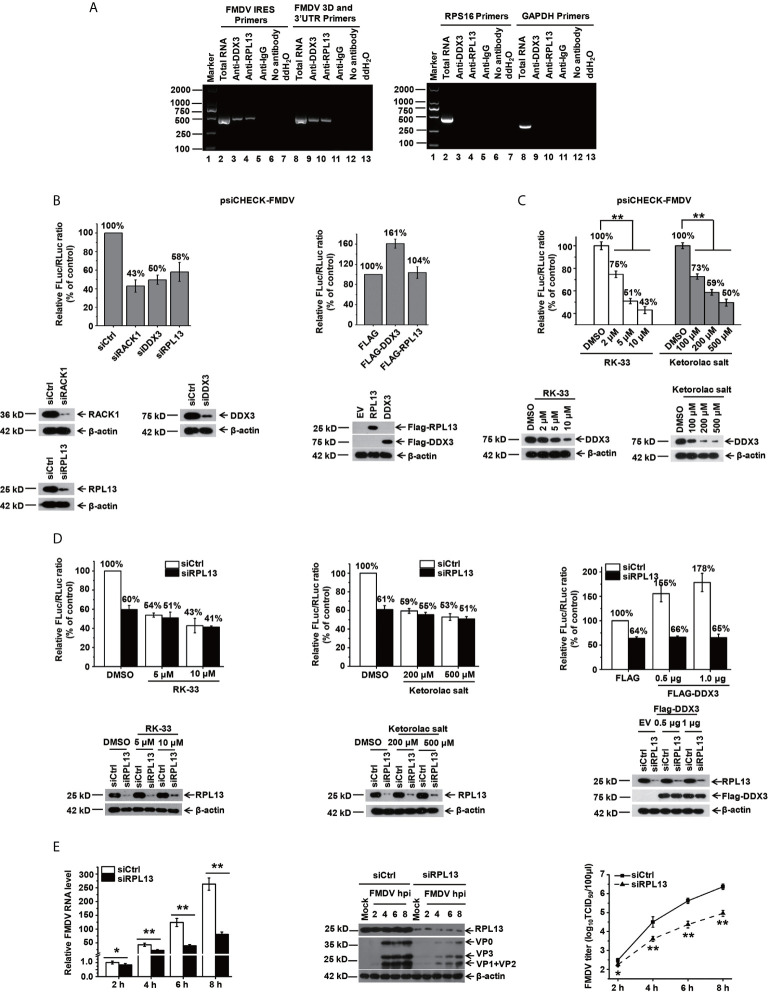
DDX3 cooperates with RPL13 to promote the IRES-dependent activity and replication of FMDV in PK-15 cells. **(A)** FMDV-infected PK-15 cell lysates at 5 hpi (MOI= 1) were subjected to an immunoprecipitation assay with an anti-RPL13 or anti-DDX3 antibody. After the cells were washed and dissociated, RNA was extracted from them and analyzed with RT–PCR using primers specific for the FMDV IRES or 3D/3′UTR, RPS16, or GAPDH. **(B)** PK-15 cells transfected with either the indicated siRNA or FLAG-tagged plasmid were transfected with the bicistronic construct psiCHECK–FMDV. At 24 h posttransfection, the RLuc and FLuc activities were measured. **(C)** PK-15 cells pretreated with the indicated amounts of RK-33 and ketorolac salt for 30 h were transfected with bicistronic plasmid psiCHECK–FMDV for 24 h in the presence of the indicated inhibitor. The cell lysates were then subjected to a luciferase reporter assay. **(D)** Control and RPL13-depleted PK-15 cells were transfected with bicistronic plasmid psiCHECK–FMDV in the presence of the indicated concentrations of RK-33 and ketorolac salt. siRNA-treated cells were transfected with the empty FLAG vector or increasing amounts of plasmid encoding FLAG–DDX3 for 24 h, and then transfected with bicistronic plasmid psiCHECK-FMDV. The RLuc and FLuc activities were determined at 24 h posttransfection. **(E)** PK-15 cells were transfected with control siRNA (siCtrl) or RPL13-targeting siRNA (siRPL13) for 48 h, and then challenged with FMDV (MOI = 0.5). At various time points, the viral RNA levels were determined with RT–qPCR, the viral proteins were detected with western blotting, and the viral titers were determined with a TCID_50_ assay. The data reflect the mean SD of three separate trials (*p < 0.05, **p < 0.01).

In our previous study, we demonstrated that when BHK-21 cells are infected with FMDV, RPL13, a molecule downstream from DEAD box helicase 3 (DDX3), synergistically promotes FMDV IRES-dependent translation and viral replication (25). In the present study, we further investigated the function of RPL13 in PK-15 cells during FMDV infection. We first examined whether viral IRES-dependent translation mediated by DDX3 and RPL13 is required when FMDV replication in PK-15 cells. Our results show that in FMDV-infected PK-15 cells, RPL13 and DDX3 interact with the FMDV genome, but do not bind to the host RPS16 and GAPDH RNA (**Figure 1A**). The knockdown of RPL13 and DDX3 inhibited FMDV IRES-dependent translation activity in PK-15 cells, whereas the overexpression of DDX3 promoted FMDV IRES activity. However, the overexpression of RPL13 had no effect on IRES activity (**Figure 1B**). In RPL13-silenced PK-15 cells, there was no significant change in FMDV IRES activity after treatment with the specific DDX3 inhibitors RK-33 and ketorolac salt, whereas the knockdown of RPL13 significantly inhibited the stimulatory effect of DDX3 on IRES activity (**Figures 1C, D**). The knockdown of RPL13 significantly inhibited the replication of FMDV in PK-15 cells, the viral RNA and protein levels, and the viral titer decreased significantly (**Figure 1E**). These results suggest that the IRES-dependent translation activity of FMDV requires the synergistic participation of DDX3 and RPL13 in the PK-15 cell line.

### Overexpression of RPL13 Inhibits FMDV Replication in PK-15 Cells

**Figure 2:**
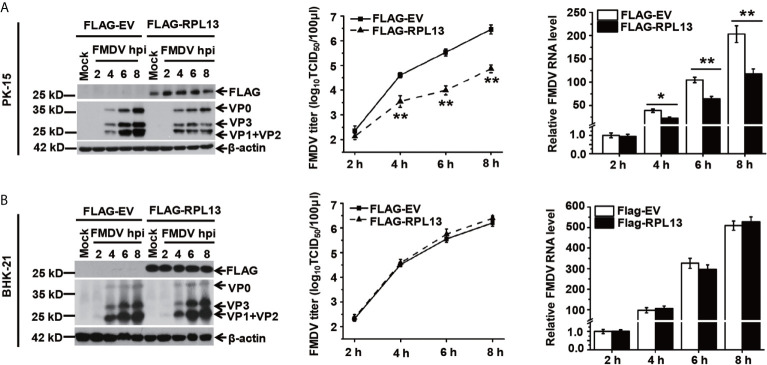
Effects of RPL13 overexpression on FMDV replication. PK-15 **(A)** and BHK-21 cells **(B)** were transfected with plasmid encoding FLAG–EV or FLAG–RPL13 for 24 h, and then challenged with FMDV (MOI = 0.5). At various time points, the viral proteins were detected with western blotting, the viral RNA levels were determined with RT–qPCR, and the viral titers were determined with a TCID_50_ assay. The data reflect the mean SD of three separate trials (*p < 0.05, **p < 0.01).

Several studies have shown that RPL13 also participates in the activation of the NF-κB signaling pathway. To determine whether RPL13 participates in the extraribosomal functions of the host innate immune system and its effect on FMDV replication, RPL13 was overexpressed in PK-15 cells with complete innate immune functions, which were then infected with FMDV. The replication of FMDV was measured at different time points. The overexpression of RPL13 significantly inhibited the replication of FMDV in PK-15 cells, and viral protein expression, viral RNA levels, and the viral titer were significantly lower in those cells than in the normal control cells (**Figure 2A**). However, in innate-immunity-deficient BHK-21 cells, the overexpression of RPL13 had no significant effect on the viral protein or RNA levels or the titer of FMDV, indicating that the overexpression of RPL13 did not affect the replication of FMDV (**Figure 2B**). The overexpression of RPL13 inconsistently affected FMDV replication in PK-15 and BHK-21 cells, leading us to infer that the overexpression of RPL13 promotes the activation of the innate immune pathway induced by FMDV, thus, inhibiting viral replication in PK-15 cells. However, BHK-21 is a congenitally immunodeficient cell line (26), so the overexpression of RPL13 had no effect on FMDV replication.

### RPL13 Promotes the Activation of the Innate Immune Signaling Pathway, but Independently of DDX3

**Figure 3:**
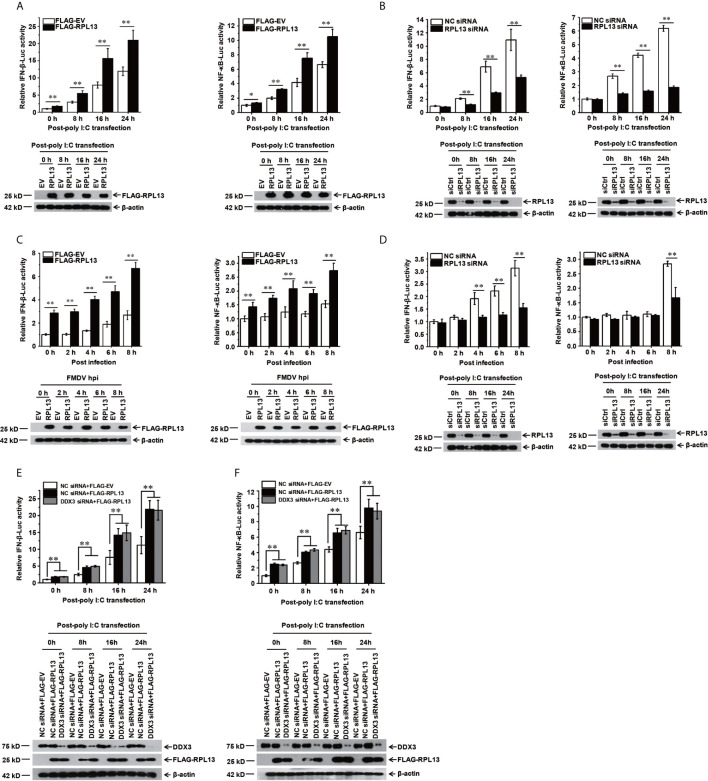
RPL13 stimulates the innate antiviral response in DDX3-independent manner. **(A–D)** PK-15 cells were cotransfected with plasmid encoding FLAG–EV or FLAG–RPL13 and pGL4–IFN-β–Luc or pGL4–NF-κB–Luc, together with the pRL-TK plasmid as the internal control. Cells treated with the control siRNA (siCtrl) or RPL13-targeting siRNA (siRPL13) for 48 h were cotransfected with a reporter plasmid and pRL-TK. At 24 h posttransfection, the cells were treated with poly(I:C) (1 μg/well) **(A, B)** or FMDV (MOI = 0.5) **(C, D)** for the indicated times. Dual luciferase activities in the cell lysates were then measured. **(E, F)** Control and DDX3-depleted PK-15 cells were cotransfected with plasmid encoding FLAG–EV or FLAG–RPL13 and pGL4–IFN-β–Luc **(E)** or pGL4–NF-κB–Luc **(F)**, together with pRL-TK plasmid. At 24 h posttransfection, the cells were treated with poly(I:C) (1 μg/well) for the indicated times. The cell lysates were then subjected to a dual luciferase reporter assay. The data reflect the mean SD of three separate trials (*p < 0.05, **p < 0.01).

To confirm that RPL13 participates in the activation of the innate immune pathway, a dual luciferase reporter assay was performed to determine the effect of RPL13 on the activation of the NF-κB and IFN-β gene promoters. We first used the IFN inducer polyinosinic:polycytidylic acid [poly(I:C)] to show that the overexpression of RPL13 in PK-15 cells significantly enhanced the poly(I:C)-induced expression of NF-κB–Luc and IFN-β–Luc (**Figure 3A**), whereas the knockdown of RPL13 in PK-15 cells significantly inhibited the poly(I:C)-induced expression of NF-κB–Luc and IFN-β–Luc (**Figure 3B**). These results suggest that RPL13 regulates the poly(I:C)-induced activation of the NF-WFI 2κB and IFN-β gene promoters. We also found that the overexpression of RPL13 promoted the expression of NF-κB–Luc and IFN-β–Luc induced by FMDV infection (**Figure 3C**), whereas the knockdown of RPL13 inhibited the FMDV-infection-induced expression of NF-κB–Luc and IFN-β–Luc (**Figure 3D**). Therefore, RPL13 mediates the activation of the innate immune pathway in PK-15 cells when stimulated by either an immune inducer or viral infection.

Because RPL13 interacts directly with DDX3 to participate in the FMDV IRES-dependent translation process and DDX3 is an important part of the antiviral immune response (25, 27), we speculated that the activation of the innate immune pathway by RPL13 involves DDX3. To test this hypothesis, DDX3-knockdown PK-15 cells were cotransfected with the FLAG-RPL13 or empty vector and plasmid encoding NF-κB–Luc or IFN-β–Luc, together with the internal reference plasmid pRL-TK. The cells were then stimulated with poly(I:C). The expression of NF-κB–Luc and IFN-β–Luc was detected at a specific time point. The results show that the overexpression of RPL13 significantly increased the poly(I:C)-induced expression of NF-κB–Luc and IFN-β–Luc, whereas the knockdown of DDX3 did not affect the stimulatory effect mediated by RPL13 (**Figures 3E, F**). These findings suggest that the activation of the antiviral immune signaling pathway mediated by RPL13 is independent of DDX3.

### RPL13 Promotes the Expression of Antiviral Factors Induced by FMDV

**Figure 4:**
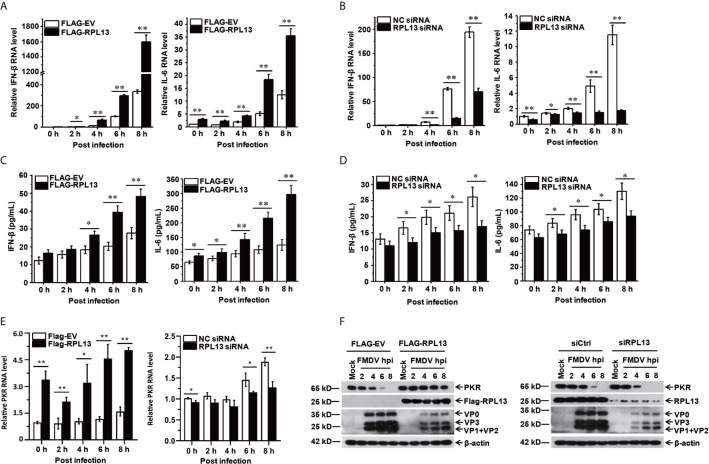
RPL13 promotes the expression of IFN-β and IL-6. PK-15 cells in which RPL13 was overexpressed or knocked down were infected with FMDV (MOI = 0.5). At the indicated times, total RNA was extracted to detect the levels of IFN-β, IL-6 and PKR mRNAs with RT–qPCR **(A, B, E)**. The cell supernatants were collected to measure IFN-β and IL-6 protein secretion with ELISAs **(C, D)**. The viral proteins were detected with western blotting **(F)**. The data reflect the mean SD of three separate trials (*p < 0.05, **p < 0.01).

To further investigate the effect of RPL13 on the FMDV-induced expression of antiviral factors, we measured the transcript and protein levels of antiviral protein IFN-β and the proinflammatory factor IL-6 induced by FMDV after the overexpression or knockdown of RPL13 in PK-15 cells. The gene and protein expression of IFN-β and IL-6 was promoted in the middle and later stages of FMDV infection. The overexpression of RPL13 significantly increased the transcript levels of IFN-β and IL-6 induced by FMDV, and the secretion of IFN-β and IL-6 proteins (**Figures 4A, C**). However, the knockdown of RPL13 significantly reduced the FMDV-induced transcription of IFN-β and IL-6 and the secretion of the proteins (**Figures 4B, D**). These results suggest that RPL13 exerts its antiviral activity by promoting the expression of cytokines such as antiviral factors IFN-β and IL-6. After FMDV infection, Type I IFN was induced and then stimulated Interferon-stimulated genes (ISGs) to inhabit virus replication. We found overexpression of RPL13 significantly increased the transcript level and protein level of the interferon-induced double-strand RNA activated protein kinase (PKR), which elicits its antiviral functions after FMDV infection. The knockdown of RPL13 had the opposite effects (**Figures 4E, F**).

### FMDV Antagonizes the Antiviral Activity of RPL13 Through Its 3C^pro^ Protease Activity

**Figure 5:**
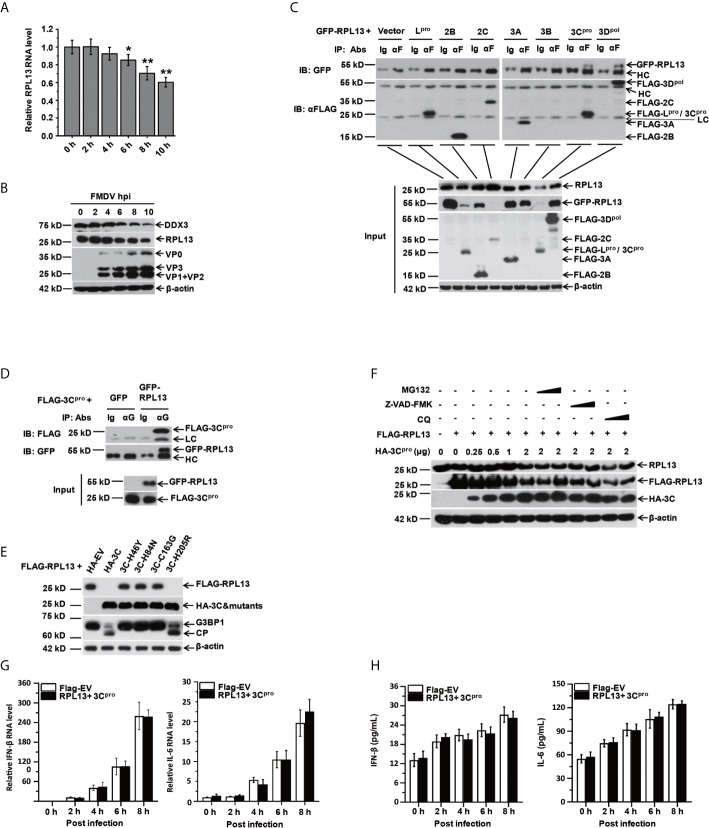
RPL13 is a specific target of FMDV proteinase 3C^pro^. **(A, B)** PK-15 cells were infected with FMDV (MOI = 0.5). At the indicated times, total RNA was extracted and subjected to qPCR analysis **(A)**, and the cell lysates were analyzed with western blotting **(B)**. **(C, D)** PK-15 cells grown in a 10 cm dish were cotransfected with plasmid encoding GFP–RPL13 (7 μg) and one of various plasmids expressing FLAG-tagged viral proteins (L^pro^, 2B, 2C, 3A, 3B, 3C^pro^, or 3D^pol^) or FLAG–EV (7 μg). At 30 h posttransfection, the cell lysates were immunoprecipitated with control IgG, anti-FLAG antibody **(C)**, or anti-GFP **(D)** antibody. The cell lysates (input) and the precipitates were then subjected to western blotting with the indicated antibodies. **(E)** PK-15 cells were cotransfected with 2 μg of plasmid encoding FLAG–RPL13 and 2 μg of plasmid encoding HA–EV, HA–3C^pro^, or various mutants of HA–3C^pro^ for 30 h. The cell lysates were subjected to western blotting with anti-FLAG, anti-HA, anti-G3BP1, or anti-β-actin antibody. **(F)** PK-15 cells were cotransfected with plasmid encoding FLAG–EV or FLAG–RPL13 and plasmid encoding HA–EV or HA–3C^pro^ in the presence or absence of MG132 (2 or 20 μM), CQ (50 or 100 μM), or Z-VAD-FMK (10 or 50 μM) for 30 h. Levels of endogenous RPL13, FLAG–RPL13, and HA–3C^pro^ was detected with western blotting. PK-15 cells in which RPL13(2μg) and HA–3C^pro^(2 μg) were cotransfected were infected with FMDV (MOI = 0.5). At the indicated times, total RNA was extracted to detect the levels of IFN-β and IL-6 mRNAs with RT–qPCR **(G)**. The cell supernatants were collected to measure IFN-β and IL-6 protein secretion with ELISAs **(H)**. The data reflect the mean SD of three separate trials ( *p < 0.05, **p < 0.01).

The results described above confirm that RPL13 enhances the antiviral immune response induced by FMDV. To test whether FMDV has evolved a mechanism to antagonize the antiviral response of RPL13 to maintain an environment favorable to viral replication, we first examined the effect of FMDV infection on the endogenous expression of RPL13. FMDV infection inhibited the gene and protein expression of RPL13 in PK-15 cells, and this inhibition increased continuously as the infection lasted (**Figures 5A, B**). We try to find if there are some small fragmentation products of RPL13, but we see nothing (data not shown). We then constructed eukaryotic expression vectors for Flag–L^pro^, Flag–2B, Flag–2C, Flag–3A, Flag–3B, Flag–3C^pro^, and Flag–3D^pol^ to determine whether RPL13 is cleaved by viral nonstructural proteins in transfected cells. The results showed that nonstructural proteins L^pro^, 2C, and 3C^pro^ significantly inhibited the expression of GFP–RPL13, but only 3C^pro^ also significantly reduced the expression of endogenous RPL13 (**Figure 5C**). Using coimmunoprecipitation with an anti-FLAG antibody, we confirmed that FLAG–3C^pro^ precipitated with GFP–RPL13, and GFP–RPL13 also precipitated with FLAG–3C^pro^ when an anti-GFP antibody was used, demonstrating the interaction between FLAG–3C^pro^ and GFP–RPL13 (**Figures 5C, D**). To verify the mechanism of RPL13 degradation by the viral protease 3C^pro^, we constructed 3C^pro^ protease mutants H46Y, H84N, C163G, and H205R, three of which (3C^pro^ H46Y, H84N, and C163G) lacked enzymatic digestion activity, whereas the enzymatic activity of 3C^pro^ H205R was intact. PK-15 cells were cotransfected with plasmid encoding FLAG–RPL13 and wild-type 3C^pro^ or the individual 3C^pro^ mutants. Wild-type 3C^pro^ and mutant H205R significantly inhibited the expression of FLAG–RPL13, whereas the loss-of-enzyme-activity 3C^pro^ mutants did not affect the expression of FLAG–RPL13 (**Figure 5E**). These findings suggest that the activity of 3C^pro^ protease is very important in the antagonism of the antiviral activity of RPL13 by FMDV. MG132 (an inhibitor of the proteasomal degradation pathway), chloroquine (an inhibitor of the lysosomal degradation pathway), and Z-VAD-FMK (an inhibitor of the apoptosis degradation pathway) were also used to treat the cells. We found that the effects of FMDV infection and 3C^pro^ protease on the degradation of endogenous and exogenous RPL13 were unrelated to the proteasomal, lysosomal, apoptotic, or other related degradation pathways (**Figure 5F**). Finally, when we overexpressed RPL13 together with FMDV 3C^pro^, we found that RPL13-induced expression of IFN-β and IL-6 was inhibited (**Figures 5G, H**). These results suggest that FMDV relies on its 3C^pro^ protease to degrade RPL13, and thus, antagonize its antiviral activity.

## Discussion

The extraribosomal functions of ribosomal proteins is a new concept put forward in recent years that has received extensive attention in the fields of oncology, immunity, and cell science. However, it has rarely been reported in the field of virology. In this study, we confirmed that RPL13 and DDX3 are required for the process of FMDV replication in the PK-15 cell line to promote the process of FMDV IRES-dependent translation, the knockdown of RPL13 significantly inhibited the replication of FMDV. Interestingly we also found that in PK-15 cells, RPL13 is also involved in the FMDV-induced antiviral immune response, inhibiting FMDV replication. The overexpression of RPL13 in PK-15 cells promoted the activation of the NF-κB and IFN-β gene promoters, and the gene expression and protein secretion of antiviral factor IFN-β and proinflammatory cytokine IL-6. On the other hand, the knockdown of RPL13 inhibited the FMDV-induced activation of the NF-κB and IFN-β gene promoters and the gene expression and protein secretion of IFN- β and IL-6. We also confirmed that the antiviral response mediated by RPL13 is independent of the helicase DDX3. However, the specific mechanism by which RPL13 regulates the FMDV-induced innate immune response requires further study. We speculated that RPL13 binds to specific transcriptional regulatory factors to promote the initiation and expression of key factors in the antiviral immune signaling pathway, or that RPL13 mediates the binding to specific secondary structures in some important nodal genes in the antiviral signaling pathway, thus promoting their expression. The expression and activation of important nodal factors in the antiviral immune pathway can initiate the expression of downstream antiviral factors and proinflammatory factors, thus exerting antiviral activity, but these speculations remain to be verified.

During its long-term evolution, FMDV has acquired a series of mechanisms with which to antagonize the antiviral activity of the host. Studies have shown that to block the activation of the innate immune system, FMDV inhibits, cleaves, or blocks some nodal molecules in the immune response signaling pathways mediated by PRRs with its own proteins, especially the FMDV nonstructural proteins L^pro^ and 3C^pro^ (28–30). Both L^pro^ and 3C^pro^ have protease activity and participate in the cleavage of FMDV polymer precursor proteins to form mature viral proteins. They also abolish host mRNA-cap-dependent translation by cleaving the host translation initiation factor eIF4G, to promote virus replication (31). Of these two proteins, L^pro^ is considered the FMDV protein most important in antagonizing the cellular innate immune response, and can directly cleave multiple nodes in a variety of innate immune pathways, thus, playing an immunosuppressive role. L^pro^ antagonizes the expression of type I IFN by cleaving NF-κB, IRF3, and IRF7, and the cleavage of IRF3/7 is known to depend on the protease activity and SAP domain of L^pro^ (32). L^pro^ also has deubiquitination activity, and inhibits the expression of IFN-β by blocking the ubiquitin activation of RIG-I, TBK1, TRAF3, and TRAF6 (29). 3C^pro^ is another important protein with which FMDV antagonizes the innate immunity of its host. 3C^pro^ inhibits the production of type I IFN by blocking the activation or cleavage of the NF-κB-regulating protein NEMO by IRF3/7 (30).

It has also been reported that FMDV nonstructural proteins 2B, 2C, and 3A and structural proteins VP1 and VP3 play immunosuppressive roles. 2B and 2C cooperate with each other or the precursor 2BC to inhibit the protein transport process by acting on the endoplasmic reticulum and Golgi apparatus and inhibiting the expression of MHC I molecules on the cell membrane, thus delaying the transition from innate immunity to acquired immunity and affecting the secretion of induced signaling molecules. The 2B protein also promotes the degradation of RIG-I, laboratory of genetics and physiology 2 (LGP2), and nucleotide-binding oligomerization domain 2 (NOD2) to counteract the antiviral activity they mediate (33). The 3A protein interacts with upstream receptor proteins RIG-I, MDA5, and adaptor VISA to block the expression of these three signaling proteins by reducing their mRNA levels, thus, preventing the production of IFN-β (34). VP1 inhibits the expression of type I IFN by interacting with the soluble drug-resistance-related calcium binding protein sorcin (35). In contrast, VP3 interacts with the VISA and inhibits its expression by interfering with VISA mRNA synthesis to prevent the production of IFN-β (36). VP3 also degrades Janus kinase 1 (JAK1) through a lysosome-dependent pathway and inhibits the IFN-γ signal transduction pathway (37). In the present study, we showed that L^pro^, 2C, and 3C^pro^ significantly inhibited the expression of GFP–RPL13, and that 3C^pro^ also significantly inhibited the expression of endogenous RPL13. 3C^pro^ interacts with RPL13, and the inhibition of RPL13 expression by 3C^pro^ depends upon its protease activity. When PK-15 cells infected with FMDV, the gene and protein expression of RPL13 all reduced, that means FMDV antagonize the antiviral activity of RPL13 is not only an important event of post-translational modification but also a transcriptional event, but the inhibition of transcription may not be caused by 3C^pro^. The mechanism FMDV acquired to antagonize the antiviral activity of RPL13 might explain why both knockdown and overexpression of RPL13 can inhabit the replication of FMDV. We speculated that because FMDV can degrade RPL13, and RPL13 can also promote IRES-dependent translation of FMDV, the amount of RPL13 in FMDV infected PK-15 cells is the most suitable for FMDV replication, that is, it can ensure that the antiviral activity of RPL13 is reduced to a minimum after RPL13 degraded, and that the remaining amount of RPL13 can meet the needs of IRES-dependent translation. The Knockdown of RPL13 breaks this balance, although the antiviral activity of RPL13 is reduced to a lower level, but it is obvious that FMDV IRES-dependent translation has a greater impact, so knockdown of RPL13 inhibited FMDV replication. It is possible that the IRES-driven translation only needs a small amount of RPL13, so overexpression of RPL13 can’t promote IRES-dependent translation, but overexpression of RPL13 will significantly enhance the antiviral activity of RPL13, so overexpression of RPL13 also inhibited the replication of FMDV. This speculation remains to be confirmed. In summary, this study provides new scientific data for an omni-directional understanding of cell antiviral defenses and the antagonistic mechanisms of viruses directed against ribosomal proteins. It also provides a new idea for the development of anti-*Picornavirus* agents.

## Data Availability Statement

The original contributions presented in the study are included in the article/supplementary material. Further inquiries can be directed to the corresponding author.

## Author Contributions

The conceptualization and design of this research was conducted by HG and SH. Experiments were mainly performed and the data was analyzed and interpreted by JG and SH. J’eW, YZ, YB, and SA also provided assistance for experiments data analysis. The original draft was prepared by JG and was reviewed and edited by HG, SS, and SH. All authors contributed to the article and approved the submitted version.

## Funding

This work was supported by the National Natural Science Foundation of China (32002272, 31873023, 32072847, 32072859) and the National Key R&D Program of China (2017YFD0500900, 760 2017YFD0502300, and 2017YFD0502200).

## Conflict of Interest

The authors declare that the research was conducted in the absence of any commercial or financial relationships that could be construed as a potential conflict of interest.

## References

[1] van Riggelen J Yetil A Felsher DW. MYC as a Regulator of Ribosome Biogenesis and Protein Synthesis. Nat Rev Cancer (2010) 10:301–9. 10.1038/nrc2819 20332779

[2] Zhou X Liao WJ Liao JM Liao P Lu H. Ribosomal Proteins: Functions Beyond the Ribosome. J Mol Cell Biol (2015) 7:92–104. 10.1093/jmcb/mjv014 25735597 PMC4481666

[3] Wan F Anderson DE Barnitz RA Snow A Bidere N Zheng L. Ribosomal Protein S3: A KH Domain Subunit in NF-kappaB Complexes That Mediates Selective Gene Regulation. Cell (2007) 131:927–39. 10.1016/j.cell.2007.10.009 18045535

[4] Sen N Paul BD Gadalla MM Mustafa AK Sen T Xu R. Hydrogen Sulfide-Linked Sulfhydration of NF-kappaB Mediates its Antiapoptotic Actions. Mol Cell (2012) 45:13–24. 10.1016/j.molcel.2011.10.021 22244329 PMC3261430

[5] Wan F Weaver A Gao X Bern M Hardwidge PR Lenardo MJ. Ikkbeta Phosphorylation Regulates RPS3 Nuclear Translocation and NF-kappaB Function During Infection With Escherichia Coli Strain O157:H7. Nat Immunol (2011) 12:335–43. 10.1038/ni.2007 PMC3062687 21399639

[6] Yang ZY Qu Y Zhang Q Wei M Liu CX Chen XH. Knockdown of Metallopanstimulin-1 Inhibits NF-kappaB Signaling At Different Levels: The Role of Apoptosis Induction of Gastric Cancer Cells. Int J Cancer (2012) 130:2761–70. 10.1002/ijc.26331 21796632

[7] Zhang HX Liu ZX Sun YP Zhu J Lu SY Liu XS. Rig-I Regulates NF-kappaB Activity Through Binding to Nf-kappab1 3’-UTR mRNA. Proc Natl Acad Sci USA (2013) 110:6459–64. 10.1073/pnas.1304432110 PMC3631665 23553835

[8] Basu A Poddar D Robinet P Smith JD Febbraio M Baldwin WM 3rd. Ribosomal Protein L13a Deficiency in Macrophages Promotes Atherosclerosis by Limiting Translation Control-Dependent Retardation of Inflammation. Arterioscler Thromb Vasc Biol (2014) 34:533–42. 10.1161/ATVBAHA.113.302573 PMC3954853 24436370

[9] Poddar D Basu A Baldwin WM 3rd Kondratov RV Barik S Mazumder B. An Extraribosomal Function of Ribosomal Protein L13a in Macrophages Resolves Inflammation. J Immunol (2013) 190:3600–12. 10.4049/jimmunol.1201933 PMC3608820 23460747

[10] Landry DM Hertz MI Thompson SR. RPS25 is Essential for Translation Initiation by the Dicistroviridae and Hepatitis C Viral Iress. Genes Dev (2009) 23:2753–64. 10.1101/gad.1832209 PMC2788332 19952110

[11] Huang JY Su WC Jeng KS Chang TH Lai MM. Attenuation of 40S Ribosomal Subunit Abundance Differentially Affects Host and HCV Translation and Suppresses HCV Replication. PLoS Pathog (2012) 8:e1002766. 10.1371/journal.ppat.1002766 22792060 PMC3394201

[12] Hertz MI Landry DM Willis AE Luo G Thompson SR. Ribosomal Protein S25 Dependency Reveals a Common Mechanism for Diverse Internal Ribosome Entry Sites and Ribosome Shunting. Mol Cell Biol (2012) 33:1016–26. 10.1128/mcb.00879-12 PMC3623076 23275440

[13] Bhat P Shwetha S Sharma DK Joseph AP Srinivasan N and Das S. The Beta Hairpin Structure Within Ribosomal Protein S5 Mediates Interplay Between Domains II and IV and Regulates HCV IRES Function. Nucleic Acids Res (2015) 43:2888–901. 10.1093/nar/gkv110 PMC4357715 25712089

[14] Haque A Mir MA. Interaction of Hantavirus Nucleocapsid Protein With Ribosomal Protein S19. J Virol (2010) 84:12450–3. 10.1128/JVI.01388-10 PMC2976419 20844026

[15] Cheng E Haque A Rimmer MA Hussein IT Sheema S Little A. Characterization of the Interaction Between Hantavirus Nucleocapsid Protein (N) and Ribosomal Protein S19 (Rps19). J Biol Chem (2011) 286:11814–24. 10.1074/jbc.M110.210179 PMC3064232 21296889

[16] Cervantes-Salazar M Angel-Ambrocio AH Soto-Acosta R Bautista-Carbajal P Hurtado-Monzon AM Alcaraz-Estrada SL. Dengue Virus NS1 Protein Interacts With the Ribosomal Protein RPL18: This Interaction is Required for Viral Translation and Replication in Huh-7 Cells. Virology (2015) 484:113–26. 10.1016/j.virol.2015.05.017 26092250

[17] Shen CL Liu CD You RI Ching YH Liang J Ke L. Ribosome Protein L4 is Essential for Epstein-Barr Virus Nuclear Antigen 1 Function. Proc Natl Acad Sci USA (2016) 113:2229–34. 10.1073/pnas.1525444113 PMC4776490 26858444

[18] Beyer AR Bann DV Rice B Pultz IS Kane M Goff SP. Nucleolar Trafficking of the Mouse Mammary Tumor Virus Gag Protein Induced by Interaction With Ribosomal Protein L9. J Virol (2013) 87:1069–82. 10.1128/JVI.02463-12 PMC3554096 23135726

[19] Mazumder B Poddar D Basu A Kour R Verbovetskaya V Barik S. Extraribosomal l13a is a Specific Innate Immune Factor for Antiviral Defense. J Virol (2014) 88:9100–10. 10.1128/JVI.01129-14 PMC4136244 24899178

[20] Li Y Dong W Shi Y Deng F Chen X Wan C. Rabies Virus Phosphoprotein Interacts With Ribosomal Protein L9 and Affects Rabies Virus Replication. Virology (2016) 488:216–24. 10.1016/j.virol.2015.11.018 26655239

[21] Yang EJ Seo JW Choi IH. Ribosomal Protein L19 and L22 Modulate TLR3 Signaling. Immune Netw (2011) 11:155–62. 10.4110/in.2011.11.3.155 PMC3153667 21860608

[22] Dong H Liu P Bai M Wang K Feng R Zhu D. Structural and Molecular Basis for Foot-and-Mouth Disease Virus Neutralization by Two Potent Protective Antibodies. Protein Cell (2021) bioRxiv2020.2012.2031.424923. 10.1007/s13238-021-00828-9 PMC9095805 33599962

[23] Shuai K Liu B. Regulation of JAK-STAT Signalling in the Immune System. Nat Rev Immunol (2003) 3:900–11. 10.1038/nri1226 14668806

[24] Fan X Han S Yan D Gao Y Wei Y Liu X. Foot-and-Mouth Disease Virus Infection Suppresses Autophagy and NF-кB Antiviral Responses Via Degradation of ATG5-ATG12 by 3C(Pro). Cell Death Dis (2017) 8:e2561. 10.1038/cddis.2016.489 PMC5386389 28102839

[25] Han S Sun S Li P Liu Q Zhang Z Dong H. Ribosomal Protein L13 Promotes Ires-Driven Translation of Foot-and-Mouth Disease Virus in a Helicase Ddx3-Dependent Manner. J Virol (2020) 94:e01679–19. 10.1128/JVI.01679-19 PMC6955262 31619563

[26] Habjan M Penski N Spiegel M Weber F. T7 RNA Polymerase-Dependent and -Independent Systems for cDNA-based Rescue of Rift Valley Fever Virus. J Gen Virol (2008) 89:2157–66. 10.1099/vir.0.2008/002097-0 18753225

[27] Valiente-Echeverria F Hermoso MA Soto-Rifo R. RNA Helicase DDX3: At the Crossroad of Viral Replication and Antiviral Immunity. Rev Med Virol (2015) 25:286–99. 10.1002/rmv.1845 26174373

[28] Liu W Yang D Sun C Wang H Zhao B Zhou G. Hnrnp K is a Novel ITAF That Negatively Regulates Foot-and-Mouth Disease Virus Translation and Replication and is Antagonized by Viral 3C Protease. J Virol (2020) 9:e00803–20. 10.1128/JVI.00803-20 PMC7431795 32581104

[29] Wang D Fang L Li P Sun L Fan J Zhang Q. The Leader Proteinase of Foot-and-Mouth Disease Virus Negatively Regulates the Type I Interferon Pathway by Acting as a Viral Deubiquitinase. J Virol (2011) 85:3758–66. 10.1128/JVI.02589-10 PMC3126127 21307201

[30] Wang D Fang L Li K Zhong H Fan J Ouyang C. Foot-and-Mouth Disease Virus 3C Protease Cleaves NEMO to Impair Innate Immune Signaling. J Virol (2012) 86:9311–22. 10.1128/JVI.00722-12 PMC3416110 22718831

[31] Han SC Guo HC Sun SQ. Three-dimensional Structure of Foot-and-Mouth Disease Virus and its Biological Functions. Arch Virol (2015) 160:1–16. 10.1007/s00705-014-2278-x 25377637

[32] de Los Santos T Diaz-San Segundo F Grubman MJ. Degradation of Nuclear Factor Kappa B During Foot-and-Mouth Disease Virus Infection. J Virol (2007) 81:12803–15. 10.1128/JVI.01467-07 PMC2169123 17881445

[33] Liu H Zhu Z Xue Q Yang F Cao W Zhang K. Foot-and-Mouth Disease Virus Antagonizes Nod2-Mediated Antiviral Effects by Inhibiting Nod2 Protein Expression. J Virol (2019) 93:e00124–19. 10.1128/JVI.00124-19 PMC6532107 30894473

[34] Li D Lei C Xu Z Yang F Liu H Zhu Z. Foot-and-Mouth Disease Virus non-Structural Protein 3A Inhibits the Interferon-Beta Signaling Pathway. Sci Rep (2016) 6:21888. 10.1038/srep21888 26883855 PMC4756384

[35] Li X Wang J Liu J Li Z Wang Y Xue Y. Engagement of Soluble Resistance-Related Calcium Binding Protein (Sorcin) With Foot-and-Mouth Disease Virus (FMDV) VP1 Inhibits Type I Interferon Response in Cells. Vet Microbiol (2013) 166:35–46. 10.1016/j.vetmic.2013.04.028 23764275

[36] Li D Yang W Yang F Liu H Zhu Z Lian K. The VP3 Structural Protein of Foot-and-Mouth Disease Virus Inhibits the IFN-beta Signaling Pathway. FASEB J (2016) 30:1757–66. 10.1096/fj.15-281410 26813975

[37] Li D Wei J Yang F Liu HN Zhu ZX Cao WJ. Foot-and-Mouth Disease Virus Structural Protein VP3 Degrades Janus Kinase 1 to Inhibit IFN-gamma Signal Transduction Pathways. Cell Cycle (2016) 15:850–60. 10.1080/15384101.2016.1151584 PMC4845950 26901336

